# Prévalence de la fibrillation atriale non valvulaire et de l´accident vasculaire cérébral ischémique et facteurs associés à la fibrillation atriale non valvulaire chez les patients hypertendus: étude observationnelle à propos de 2887 patients

**DOI:** 10.11604/pamj.2021.38.31.25569

**Published:** 2021-01-13

**Authors:** Amine Bahloul, Nadia Bouattour, Faten Triki, Rania Hammami, Selma Charfeddine, Tarek Ellouze, Chokri Mhiri, Leila Abid, Samir Kammoun

**Affiliations:** 1 Service de Cardiologie, Centre Hospitalo-Universitaire Hèdi Chaker, Sfax, Tunisie,; 2 Service de Neurologie, Centre Hospitalo-Universitaire Habib Bourguiba, Sfax, Tunisie

**Keywords:** Hypertension artérielle, fibrillation atriale, accident vasculaire cérébral ischémique, accident vasculaire cérébral cardio-embolique, épidémiologie, High blood pressure, atrial fibrillation, ischemic stroke, embolic stroke of cardiac origin, epidemiology

## Abstract

Résumé

**Introduction:**

l´hypertension artérielle (HTA), la fibrillation auriculaire (FA) et l´accident vasculaire cérébral (AVC) sont trois problèmes de santé publique. A travers cette étude nous avons cherché à déterminer la prévalence de la FA et de l´AVC ischémique chez les patients hypertendus et déterminer les facteurs associés à la survenue de la FA non valvulaire chez ces patients.

**Méthodes:**

nous avons utilisé les données recueillies dans le service de cardiologie de l´hôpital universitaire de Sfax dans le cadre du registre national tunisien d´HTA. Nous avons étudié les associations entre les différentes variables sociodémographiques, cliniques, paracliniques et thérapeutiques et la FA chez les hypertendus en utilisant des modèles de régression logistique.

**Résultats:**

notre échantillon comprenait 2887 patients avec un sexe ratio à 0,95. L´âge moyen était de 65 ans (±11). La FA était notée chez 230 patients (8%), alors que l´AVC ischémique chez 152 patients (5,3%). Au décours de l´analyse multivariée, les facteurs indépendants associés à une augmentation de la prévalence de la FA étaient: l´âge avancé (p=0,001, Odds Ratio(OR) = 1,647, Intervalle de confiance (IC) à 95%: 1,1227-2,213), la présence d´une hypertrophie ventriculaire gauche (HVG) à l´échographie cardiaque(p = 0,004, OR = 2,140, IC95%: 1,281-3,576), et la fraction d´éjection du ventricule gauche (FEVG) < 50% (p < 0,001, OR = 4,677, IC95%: 2,715-8,057).

**Conclusion:**

nous avons confirmé qu´il existe une relation directe et indépendante entre l´HTA, l´âge avancé, l´HVG et la FA. L´installation d´une FA au cours de l´HTA constitue un tournant évolutif de la maladie par majoration du risque d´AVC ischémique. Un contrôle optimal de la pression artérielle tensionnelle être hautement prioritaire chez les hypertendus, en particulier âgés, afin de prévenir le risque de la FA et de l´AVC.

## Abstract (en)

**Introduction:**

high blood pressure (HBP), atrial fibrillation (AF) and ischemic stroke are three public health problems. The purpose of this study was to determine the prevalence of AF and ischemic stroke in hypertensive patients and the factors associated with the occurrence of non-valvular AF in these patients.

**Methods:**

we used data collected in the Department of Cardiology at the University Hospital of Sfax as a part of National Tunisian Registry of Hypertension. We examined the associations between different socio-demographic, clinical, paraclinical, and therapeutic variables and AF in hypertensive patients using logistic regression models.

**Results:**

our sample consisted of 2887 patients with a sex ratio of 0.95. The average age of patients was 65 years (±11). AF was found in 230 patients (8%), while ischemic stroke was found in 152 patients (5.3%). Multivariate analysis highlighted that independent factors associated with an increase in the prevalence of AF were: advanced age (p = 0,001, Odds Ratio (OR) = 1,647, 95% confidence interval ( CI): 1,1227-2,213), the detection of left ventricular hypertrophy (LVH) on cardiac ultrasound (p= 0,004, OR= 2,140, 5%CI 9: 1,281-3,576), and left ventricular ejection fraction (LVEF) < 50% (p<0,001, OR=4,677, 95% CI: 2,715 and 8.057).

**Conclusion:**

this study confirms that there is a direct and independent relationship between high blood pressure, advanced age, LVH and AF. AF in patients with high blood pressure is a disease progression leading to an increased risk of ischemic stroke. Optimal high blood pressure control is a priority in hypertensive patients, especially in the elderly, aiming at preventing the risk of AF and ischemic stroke.

## Introduction

L´hypertension (HTA), la fibrillation auriculaire (FA) et l´accident vasculaire cérébral (AVC) sont trois problèmes de santé publique. Leurs prévalences augmentent dans le monde entier et les trois pathologies coexistent souvent chez le même patient [1]. La prévalence de la FA dans la population générale est estimée entre 1 et 2% [2]. Elle augmente chez les patients présentant certains facteurs de risque tels que l´HTA qui est présente chez environ deux tiers des individus ayant des antécédents de FA [3]. La survenue d´une FA chez les hypertendus représente un véritable tournant évolutif chez ces patients en raison des complications thromboemboliques, en particulier les AVC ischémiques [4]. Il existe des données limitées sur la prévalence de la FA et l´AVC en Tunisie, à la fois dans la population générale et chez les patients ayant des facteurs de risque tels que l´HTA. A travers cette étude nous avons cherché à déterminer la prévalence de la FA et de l´AVC chez les patients hypertendus et déterminer les facteurs associés a` la survenue de la FA non valvulaire chez ces patients.

## Méthodes

**Type et cadre de l´étude:** pour répondre à notre objectif, nous avons utilisé une partie des données recueillies dans le service de cardiologie de l´hôpital universitaire de Sfax entre le 15 avril 2019 et le 15 mai 2019 dans le cadre du registre national tunisien d´hypertension artérielle (NATURE HTN). Le registre NATURE HTN est une étude nationale, observationnelle, transversale et multicentrique planifiée par la Société Tunisienne de Cardiologie et Chirurgie Cardiovasculaire et validée par un comité d´éthique. Il a été conçu par un comité multidisciplinaire de cardiologues de différentes structures hospitalières en Tunisie.

**Population de l´étude:** ce registre a recruté tous les patients hypertendus connus ayant consulté leur médecin pendant un mois (15 avril 2019-15 mai 2019) et ayant pour principaux objectifs de décrire le profil épidémiologique de l´HTA en Tunisie. Les critères d´inclusion étaient un âge de plus de 18 ans avec une HTA essentielle confirmée. Les patients ayant une espérance de vie courte et une insuffisance rénale terminale sous hémodialyse chronique ont été exclus.

### Recueil des données

**La pression artérielle:** la PA systolique (PAS) et la PA diastolique (PAD) ont été mesurées à l´aide d´un sphygmomanomètre manuel ou automatisé conformément aux recommandations de la Société Européenne de cardiologie (ESC) et la Société européenne d´HTA (ESH) [5] sur le bras droit à l´aide d´un brassard adapté à la circonférence du bras. Les mesures ont été effectuées après 15 minutes de repos, en position assise, le bras placé dans la position appropriée. Trois mesures ont été effectuées à 1 à 2 minutes d´intervalle. Les PAS et PAD utilisées pour chaque patient correspondent à la moyenne des deux dernières mesures. Les patients qui n´ont pas eu au moins deux lectures de PA ont été exclus de l´analyse. Nous avons considéré que l´HTA est non contrôlée si la PAS est supérieure ou égale à 140 mmHg et la PAD est supérieure ou égale à 90 mmHg [5].

**Les covariables:** nous avons collecté, à travers un questionnaire, les données démographiques (l´âge et le sexe), le poids et la taille avec calcul de l´indice de masse corporelle (IMC), les comportements hygiéno-diététiques (le respect ou non du régime hyposodé, la pratique ou non d´une activité physique, le statut tabagique: fumeur actuel ou non-fumeur, et l´observance au traitement antihypertenseurs), le niveau d´éducation, les antécédents médicaux (antécédents de diabète sucré, dyslipidémie, maladie coronaire, accident vasculaire cérébral (AVC), et les données thérapeutiques (nombre et classes des médicaments antihypertenseurs à savoir un inhibiteur de l´enzyme de conversion de l´angiotensine (IEC), un antagoniste du récepteur à l´angiotensine II (ARA II), un inhibiteurs calcique, un diurétique, un bétabloquant (BB)). La présence ou non d´une FA a été cherché par l´interrogatoire, l´examen clinique et l´électrocardiogramme (ECG). La recherche d´une insuffisance rénale, définie par une clairance de la créatinine < 60/mn calculée selon la formule MDRD, a été effectué sur la base de la dernière valeur de créatinine rapportée par le patient et datant de moins de 6 mois. La recherche d´une hypertrophie ventriculaire gauche (HVG) a été effectué sur le ECG réalisé datant de moins de 6 mois (définie par un indice de Sokolow = 35 mm) et sur la dernière échocardiographie réalisée datant de moins de 12 mois (définie par une masse ventriculaire gauche > 95 g/m^2^ chez la femme et > 115g/m^2^ chez l´homme). Nous avons recueilli la fonction ventriculaire gauche (FEVG) chez les patients qui avaient une échocardiographie récente, datant de moins de 12 mois. En ce qui concerne le regroupement, nous avons divisé l´âge en 4 groupes: < 50 ans, 50-59 ans, 60-69 ans, et > 70 ans. Une catégorisation de l´IMC a été faite: < 30 kg / m^2^ comme non obèse, et = 30 kg / m^2^ comme obèse. L´ancienneté de l´HTA est divisée en HTA récente (= 1an) et ancienne (> 1 an). La FEVG a été divisée en 2 groupes (< 50% et = 50%). Nous avons classé le niveau d´éducation en quatre groupes: pas d´enseignement, école primaire, lycée secondaire et université.

**Analyse statistique:** les données recueillies ont été saisies et analysées par SPSS 23.0, qui a permis de réaliser l´ensemble des analyses statistiques. Nous avons réalisé initialement une analyse descriptive de cette population en comparant les deux sexes puis une analyse univariée pour étudier les relations entre la FA et les variables sociodémographiques, cliniques, paracliniques et thérapeutiques. Nous avons ensuite réalisé une analyse multivariée pour chercher des associations indépendantes entre la FA et les différentes variables étudiées en incluant dans le modèle statistique la classe d´âge, le sexe, et toutes les variables ayant un indice de significativité (p) < 0,2. Les données continues ont été présentées sous forme de moyenne et écart type alors que les variables catégorielles étaient présentées par des nombres et des pourcentages. La comparaison des pourcentages sur séries indépendantes a été effectuée par le test du chi 2 de Pearson et la comparaison des données continues a été effectuée par le test de Student. Nous avons utilisé pour les analyses multivariées des modèles de régression logistique. Une valeur de l´indice de significativité (p) inférieure à 0,05 était considérée comme significative.

**Considérations éthiques:** cette étude a été conduite après avoir obtenu l´autorisation du comité national d´éthique médicale. Les aspects éthiques ont été respectés. Le consentement éclairé des patients a été requis pour tous les participants à l´étude. La participation à l´étude était gratuite. La confidentialité et l´anonymat ont été garantis aux patients qui ont participé à l´étude. L´étude n´a pas exposé les patients à des risques additionnels.

## Résultats

**Prévalence de la FA et de l´AVC chez les hypertendus et caractéristiques générales de la population:** notre échantillon comprenait 2887 patients avec un sexe ratio à 0,95. L´âge moyen était de 65 ans (±11). La FA était notée chez 230 patients (8%), alors que les antécédents d´AVC ischémique étaient notés chez 152 patients (5,3%). Les facteurs de risque cardiovasculaires les plus associés à l´HTA étaient la dyslipidémie avec une prédominance féminine suivie du diabète à répartition égale entre les 2 sexes. La majorité des patients inclus étaient à faible niveau d´instruction (illettrés ou niveau primaire) avec un niveau plus élevé pour les hommes (Tableau 1).

**Tableau 1:** caractéristiques générales de la population

		Sexe		p	Total
		Homme N (%)	Femme N (%)		
**Caractéristiques cliniques**	Diabète	548 (38,9)	608 (41,1)	0,254	1156 (40,0)
	Dyslipidémie	660 (46,9)	591 (39,9)	<0,001	1251 (43,3)
	Obï¿½sité IMC>30Kg/m^2^)	248 (17,6)	544 (36,8)	<0,001	794 (27,5)
	AVC	104 (7,4)	91 (6,1)	0,207	195 (6,8)
	AVC ischémique	82 (5,8)	70 (4,7)	0,187	152 (5,3)
	AVC hémorragique	12 (0,9)	10 (0,7)	0,584	22
	Coronaropathie	419 (29,8)	211 (14,3)	<0,001	630 (21,8)
	Tabagisme	368 (26,2)	22 (1,5)	<0,001	390 (13,5)
**Niveau scolaire**	Pas d´enseignement	133 (15,5)	440 (46,4)	<0,001	573 (31,7)
	école primaire	303 (35,3)	275 (29)		578 (32,0)
	Lycée	279 (32,5)	164 (17,3)		443 (24,5)
	Université	144 (67,6)	69 (7,3)		213 (11,8)
**Caractéristiques paracliniques**	HVG à l´ ECG	213 (15,1)	169 (11,4)	0,04	382 (13,2)
	HVG à l´échographie cardiaque	227 (16,1)	198 (13,4)	0.04	425 (14,7)
	FEVG < 50%	140 (21,4)	78 (12,2)	<0,001	218 (16,9)
	Micro-albuminurie positive	37 (2,6)	31 (2,1)	0,29	68 (2,4)
	IRC (clairance <60 ml/mn)	82 (5,8)	65 (4,4)	0,09	147 (5,1)
**Nombres de médicaments antihypertenseurs**	Aucun médicament	171 (12,2)	148 (10)	0,32	319 (11,1)
	Monothérapie	724 (51,5)	776 (52,4)		1500 (52)
	Bithérapie	365 (25,9)	392 (26,5)		757 (26,2)
	Trithérapie	147 (10,4)	164 (11,1)		311 (10,8)
**Classe thérapeutique**	IEC	728 (51,7)	770 (52)	0,878	1498 (51,9)
	ARA II	266 (18,9)	286 (19,3)	0,775	552 (19,1)
	IC	499 (35,5)	492 (33,2)	0,209	991 (34,3)
	Diurétique	171 (12,2)	203 (13,7)	0,211	374 (13)
	BB	280 (19,9)	283 (19,1)	0,598	563 (19,5)

ARA II: antagonistes du récepteur de l´angiotensine II, BB: bêtabloquants, AVC: accident vasculaire cérébral, ECG: électrocardiogramme, FEVG: fraction d´éjection du ventricule gauche, HVG: hypertrophie ventriculaire gauche, IEC: inhibiteur de l´enzyme de conversion

**Statut tensionnel de la population et traitement anti-hypertenseur:** plus de la moitié des patients hypertendus étaient équilibrée (53,3%). La valeur moyenne de la pression artérielle systolique (PAS) était égale à 138,33 mmHg (±19,86 mmHg) alors que la valeur moyenne de la pression artérielle diastolique (PAD) était égale à 78,87 mmHg (±11,46 mmHg). Plus de la moitié des patients étaient traités par une monothérapie (52%). Les inhibiteurs de l´enzyme de conversion de l´angiotensine (IEC) étaient les médicaments les plus prescrits (51,9%), plus particulièrement le Captopril (69% des prescriptions par IEC).

**Facteurs sociodémographiques, cliniques, para cliniques et thérapeutiques associés à la FA chez les hypertendus:** à travers une analyse univariée puis multivariée (Tableau 2). Nous avons trouvé comme facteurs associés de façon significative et indépendante à une augmentation de la prévalence de la FA: l´âge avancé (p = 0,001, Odds Ratio (OR) =1,647, intervalle de confiance( IC) à 95%: 1,1227-2,213), la présence d´une hypertrophie ventriculaire gauche (HVG) à l´échographie cardiaque (p= 0,004, OR= 2,140, IC 95%: 1,281-3,576), et la fraction d´éjection du ventricule gauche (FEVG) < 50% (p < 0,001, OR = 4,677, IC95%: 2,715-8,057). En revanche, nous avons trouvé une diminution significative et indépendante de la prévalence de la FA chez les hypertendus tabagiques (prévalence = 3,8%, p= 0,044, OR= 0,359, IC 95%: 0,132-0,972), et les hypertendus ayant des antécédents de maladie coronaire (prévalence = 6,5%, p < 0,001, OR= 0,321, IC 95%: 0,175-0,592).

**Tableau 2:** analyse univariée et multivariée des facteurs associés à la FA chez les hypertendus

		N (%)	Analyse univariée		Analyse multivariée	
Variables étudiées			nOR IC 95%	p	aOR IC 95%	p
Classe d´ âge (ans)	<50	6 (2)	1,832 (1,555-2,158)	<0,001	1,647 (1,227-2,213)	0,001
	[50-60[	35 (4,8)				
	[60-70[	71 (7,3)				
	>70	117 (13,3)				
Sexe	Masculin	92 (6,5)	1,470 (1,117-1,934)	0,006	1,034 (0,613-1,742)	0,900
	Féminin	138 (9,3)				
Diabète		91 (7,9)	0,977 (0,74 -1,287)	0,88	0,805 (0,494-1,312)	0,385
HTA ancienne (> 1 an)		179 (9,5)	2,062 (0,967-4,295)	0,048	1,511 (0,540-4,228)	0,432
Objectif non atteint (PA ≥140/90 mmHg)		100 (7,4)	1,15 (0,897-1,509)	0,17	1,100 (0,700-1,730)	0,679
Dyslipidémie		113 (9)	1,287 (0,983-1,685)	0,071	1,441 (0,881-2,356)	0,146
Obésité (IMC>30 Kg/m^2^)		70 (8,8)	1,169 (0,872-1,568)	0,31	1,475 (0,899-2,420)	0,124
Maladie coronaire		41 (6,5)	0,76 (0,536-1,079)	0,13	0,321 (0,175-0,592)	<0,001
Hypothyroïdie		14 (13,9)	1,916 (1,072-3,426)	0,037	1,487 (0,518-4,272)	0,461
Tabac		15 (3,8)	0,425 (0,249 -0,725)	0,001	0,359 (0,132-0,972)	0,044
Niveau d´ éducation	Pas d´enseignement	89 (15,5)	0,636 (0,532-0,761)	<0,001	0,859 (0,648 -1,139)	0,292
	Primaire	38 (6,6)				
	Lycée	24 (5,4)				
	Universitaire	15 (7)				
HVG ECG		53 (13,9)	2,121 (1,528-2,943)	<0,001	0,916 (0,526 -1,595)	0,756
HVG à l´ échographie		70 (16,6)	2,83 (2,092-3,829)	<0,001	2,140 (1,281-3,576)	0,004
FEVG<50%		44 (20,2)	2,67 (1,806-3,964)	<0,001	4,677 (2,715-8,057)	<0,001
Micro-albuminurie positive		6 (8,8}		0,36		
IRC (clairance <60 ml/mn)		17 (11,6)	1,553 (0,919-2,623)	0,11	0,663 (0,261-1,688)	0,389
Thérapie (nombre d´antihypertenseurs)	Mono	102 (6,8)	1,427 (1,217-1,647)	<0,001	1,003 (0,722-1,395)	0,985
	Bithérapie	74 (9,8)				
	Trithérapie	39 (12,5)				
IEC		71 (7,2)		0,37		
ARA II		50 (9)		0,29		
Inhibiteurs calciques		71 (7,2)		0,27		
Diurétique		43 (11,5)	1,612 (1,135-2,289)	0,01	0,899 (0,504-1,605)	0,720
BB		69 (12,2)	1,874 (1,390-2,525)	<0,001	1,790 (0,958-3,344)	0,068

aOR: Odds Ratio ajusté, ARA II: antagonistes du récepteur de l´angiotensine II, BB: bêtabloquants, ECG: électrocardiogramme, FA: fibrillation atriale, FEVG: fraction d´ éjection du ventricule gauche, HTA: hypertension artérielle, HVG: hypertrophie ventriculaire gauche, IC: intervalle de confiance à 95%, IEC: inhibiteurs de l´enzyme de conversion de l´angiotensine, IMC: indice de masse corporelle, IRC: insuffisance rénale chronique, N(%): nombre et pourcentage des patients ayant une fibrillation auriculaire, nOR:Odds Ratio non ajusté, PA: pression artérielle

## Discussion

L´HTA constitue un facteur de risque puissant et indépendant de survenue de FA non valvulaire [4]. La prévalence de la FA dans la population générale est estimée entre 1 et 2% [2, 6]. Conformément à ce qui a été trouvé dans notre étude, la prévalence augmente chez les patients présentant certains facteurs de risque tels que l´HTA [3, 7]. Elle varie de 3,5% à 13% chez les patients hypertendus selon les études [8-11]. Dans l´analyse univariée, nous avons trouvé comme prévu les facteurs associés à une augmentation de la prévalence de la FA à savoir l´âge avancé, l´HTA ancienne, le bas niveau d´éducation, l´hypothyroïdie, la présence d´une HVG à l´ECG et/ou à l´échographie cardiaque, la FEVG < 50% et nombre élevé de médicaments antihypertenseurs. Cependant dans l´analyse multivariée, seulement l´âge avancé, l´HVG à l´échographie cardiaque et la FEVG <50% étaient associés à une augmentation significative et indépendante de la prévalence de la FA.

Les résultats de notre étude concernant l´association entre l´âge avancé et la FA étaient similaires à ceux trouvées dans les études antérieurs [3, 11, 12]. La prévalence de la FA augmente avec l´âge en raison du processus dégénératif du muscle auriculaire et des cellules conductrices [7]. Quant à l´effet du sexe, contrairement aux résultats trouvées dans les études antérieurs [1, 4] qui ont trouvé une prévalence plus élevée de la FA chez les hommes, nous avons trouvé une prédominance féminine qui s´atténue après ajustement aux variables cliniques, paracliniques et thérapeutiques. Contrairement aux résultats trouvés dans la littérature [13, 14], le mauvais contrôle de la PA n´était pas associé à une augmentation de la prévalence de la FA dans notre étude. En revanche le traitement par une trithérapie ou plus, traduisant souvent la difficulté d´équilibrer la PA chez ces patients, était associé à une prévalence plus élevée de la FA dans l´analyse univariée. Dans une étude de population cas-témoins, le risque de FA a doublé chez les individus avec PAS = 150 mmHg, par rapport aux patients avec des niveaux de PAS de 120 à 129 mmHg [13]. En revanche, l´impact de la réduction intensive de la PA sur le risque de FA est encore indéfini en raison de la rareté des données publiées.

Le rôle du tabagisme est discuté, l´augmentation du risque de fibrillation atriale semble être faible dans certaines études [15, 16], alors que d´autres études n´ont trouvé aucune association [17]. Bien que le tabagisme augmente tous les mécanismes potentiellement impliqués dans l´étiologie de la FA dont le stress oxydatif, l´inflammation et la fibrose auriculaire, ces données incohérentes ainsi que les résultats de notre étude montrant une effet négatif significatif et indépendant du tabagisme sur le risque de FA chez les patients hypertendus, suggèrent que des études plus approfondies sur l´association entre le tabagisme et la FA sont nécessaires. Notre étude n´a pas montré aussi une association significative entre l´obésité et la FA contrairement à ce qui a été décrit dans plusieurs études dans lesquelles l´obésité est reconnue comme facteur de risque indépendant de FA [18, 19]. Les mécanismes physiopathologiques reliant l´obésité et la FA sont très complexes .Ils incluent une dérégulation hémodynamique, neuro-humorale, métabolique et inflammatoire [20]. Les résultats des études qui ont étudié l´association entre l´hypothyroïdie et la FA sont contrastés. Certaines n´ont pas trouvé une association significative [21, 22] tel est le cas dans notre étude, d´autres ont trouvé une association positive [23, 24].

Dans notre étude nous avons trouvé une association significative entre le niveau d´éducation et la FA seulement dans l´analyse univariée qui s´atténue après ajustement aux autres facteurs cliniques et thérapeutiques. Le niveau d´éducation est décrit comme facteur prédictif indépendant de la FA dans plusieurs études: un bas niveau d´éducation est souvent associé à un mauvais équilibre tensionnel augmentant ainsi le risque de survenue de FA [25]. L´HVG détectée à l´ECG ou mieux à l´échocardiographie, est un facteur de risque important et puissant de FA chez les sujets hypertendus [26]. Un résultat qui va de pair avec ce qui a été trouvé dans notre étude. L´HVG est la conséquence de l´adaptation myocardique à la surcharge de pression. Dans l´étude Framingham, les patients ayant une HVG électrique avaient au moins 3 fois plus de risque de développer une FA [27].

Nos résultats mettent en évidence une relation étroite entre la FA et la fonction ventriculaire gauche chez les patients hypertendus. Nous avons trouvé qu´une FEVG < 50% était fortement associée à une prévalence plus élevée de FA (p < 0,001, OR = 4,677, IC95%: 2,715-8,057). Cette association entre la diminution de la FEVG et la FA a été démontrée par de nombreuses études. Chacune de ces deux conditions prédispose à l´autre, et lorsqu´elles sont présentes en association, elles sont associées à un plus mauvais pronostic que l´une ou l´autre condition seule [28]. En revanche, la présence d´une maladie coronaire chez les hypertendus était associée à une prévalence moins élevée de FA (p < 0,001, OR = 0,321). Cette relation entre la FA et la maladie coronaire, apparue uniquement dans l´analyse multivariée, pourrait s´expliquer par une prise en charge thérapeutique plus intensive de l´HTA chez ces patients, diminuant ainsi le risque d´apparition de la FA.

Nous n´avons pas trouvé un effet significatif du type du traitement sur la prévalence de la FA malgré que plusieurs études suggèrent que les bloqueurs du système rénine-angiotensine-aldostérone pourraient réduire l´incidence de la FA indépendamment du niveau tensionnel [29]. Cet effet anti-arythmique de cette classe médicamenteuse demande, cependant, d´avantage de confirmation. La survenue d´une FA chez les hypertendus représente un véritable tournant évolutif chez ces patients en raison des complications emboliques, en particulier Les AVC ischémique [4]. A ce titre, la Société Européenne de Cardiologie (ESC) identifie officiellement l´HTA comme étant un facteur de risque d´accidents thromboemboliques au cours de la FA non valvulaire, l´ESC recommande une stratification du risque thromboembolique par l´évaluation du score CHA2DS2 VASC (cardiopathie, hypertension, âge = 75 ans, diabète, AVC, pathologie vasculaire, âge entre 65 et 74 ans et le sexe féminin). Un score = 1 impose une anticoagulation orale en dehors des contre-indications (recommandation de Classe IIa) [30].

Les résultats de notre étude ont des implications cliniques. Notre objectif principal était d´obtenir une estimation précise de prévalence de la FA dans la population hypertensive. Notre estimation de prévalence de 8% de FA dans la population hypertendue est élevée compte tenu de l´évolution maligne de cette complication qui pourrait provoquer un AVC thromboembolique grave. Nous avons démontré qu´il existe d´autres facteurs qui pourraient augmenter encore la prévalence de la FA et par conséquent de l´AVC surtout lorsqu´ils sont associés. Une implication de ces résultats est que ces facteurs de risque doivent être pris en compte lors de l´élaboration de stratégies préventives. L´observation que le risque de FA est plus élevé chez les patients qui ont une HTA compliquée d´une HVG à l´ECG et/ou à l´échocardiographie appelle à un plan de prévention sur les principaux facteurs liés au mode de vie par une bonne application des mesures hygiéno-diététiques et une bonne observance du traitement antihypertenseur afin d´obtenir un meilleur contrôle tensionnel et prévenir l´apparition de l´HVG. Un bon contrôle tensionnel par modifications du mode de vie et un renforcement du traitement médical doit être hautement prioritaire chez les patients hypertendus ayant une HVG à l´ECG et/ou à l´échocardiographie afin de prévenir les événements cardiovasculaires, en particulier la FA et l´AVC.

Certaines limites de notre étude méritent d´être mentionnées. En effet, cette étude ne prévoyait pas initialement d´étudier la prévalence de la FA, et la collecte des données ECG n´était pas systématique chez tous les patients, une sous-estimation de la vraie prévalence de la FA est possible. Enfin, il s´agit d´une étude transversale et non une étude de cohorte, les données ont été analysées rétrospectivement après le début de la FA. Des informations précises sur l´état des patients avant l´apparition de la FA n´étaient pas disponibles, de ce fait les facteurs associés à la FA ne peuvent pas être conclus comme facteurs de risque, mais plutôt les facteurs associés à la FA.

## Conclusion

L´installation d´une FA chez les patients hypertendus constitue un tournant évolutif de la maladie par une majoration significative du risque d´AVC ischémique. Nous avons confirmé qu´il existe une relation directe et indépendante entre l´âge avancé, l´augmentation de la masse ventriculaire gauche, la fonction ventriculaire gauche et la survenue de FA chez ces patients. Un bon contrôle tensionnel doit être hautement prioritaire chez les hypertendus, en particulier âgés, afin de prévenir le risque de la FA et de l´AVC.

### Etat des connaissances sur le sujet

- Les prévalences de l´HTA, la FA et l´AVC ischémique augmentent dans le monde entier;
- Le risque de FA et/ou d´AVC ischémique augmente chez les patients présentant certains facteurs de risque tels que l´HTA;
- Il existe des données limitées sur la prévalence de la FA et l´AVC en Tunisie, à la fois dans la population générale et chez les patients hypertendus.

### Contribution de notre étude à la connaissance

- Nous avons démontré qu´il existe des facteurs sociodémographiques et cliniques qui pourraient augmenter la prévalence de la FA et donc de l´AVC ischémique surtout lorsqu´ils sont associés;
- Il existe une relation directe et indépendante entre l´HTA, l´augmentation de la masse du ventriculaire gauche, et la survenue de FA;
- L´établissement d´un registre national multicentrique prospectif de FA avec un échantillon plus large est nécessaire pour clarifier nos résultats.
